# A Review of Leveraging Artificial Intelligence to Predict Persistent Postoperative Opioid Use and Opioid Use Disorder and its Ethical Considerations

**DOI:** 10.1007/s11916-024-01319-2

**Published:** 2025-01-23

**Authors:** Rodney A. Gabriel, Brian H. Park, Chun-Nan Hsu, Alvaro A. Macias

**Affiliations:** 1 Division of Perioperative Informatics, Department of Anesthesiology, University of California, San Diego, La Jolla, CA USA; 2 Department of Biomedical Informatics, University of California, San Diego Health, La Jolla, CA USA; 3 Department of Neurosciences, University of California, San Diego, La Jolla, CA USA

**Keywords:** Artificial intelligence, Machine learning, Persistent opioid use, Opioid use disorder, Surgery

## Abstract

**Purpose of Review:**

Artificial intelligence (AI) offers a new frontier for aiding in the management of both acute and chronic pain, which may potentially transform opioid prescribing practices and addiction prevention strategies. In this review paper, not only do we discuss some of the current literature around predicting various opioid-related outcomes, but we also briefly point out the next steps to improve trustworthiness of these AI models prior to real-time use in clinical workflow.

**Recent Findings:**

Machine learning-based predictive models for identifying risk for persistent postoperative opioid use have been reported for spine surgery, knee arthroplasty, hip arthroplasty, arthroscopic joint surgery, outpatient surgery, and mixed surgical populations. Several machine learning-based models have been described to predict an individual’s propensity for opioid use disorder and opioid overdose. Natural language processing and large language model approaches have been described to detect opioid use disorder and persistent postsurgical opioid use from clinical notes.

**Summary:**

AI holds significant promise in enhancing the management of acute and chronic opioids, which may offer tools to help optimize dosing, predict addiction risks, and personalize pain management strategies. By harnessing the power of AI, healthcare providers can potentially improve patient outcomes, reduce the burden of opioid addiction, and contribute to solving the opioid crisis.

## Introduction

The opioid crisis has posed significant challenges to healthcare systems globally, which necessitates innovative solutions to improve pain management and reduce opioid dependency [1]. Artificial intelligence (AI), with its capabilities in predictive modeling and natural language processing, offers a new frontier in the management of both acute and chronic pain, which may potentially transform opioid prescribing practices and addiction prevention strategies. In this review, we discuss various AI approaches in predicting and identifying persistent opioid use after surgery, the development of opioid use disorder, and opioid overdoses. Two prominent domains in AI include machine learning and natural language processing. Machine learning is a method of data analysis that automates analytical model building. It is a branch of AI based on the idea that systems can learn from data, identify patterns, and make decisions with minimal human intervention. (2, 3) Deep learning is a more specialized domain within machine learning that refers to utilizing neural networks with several hidden layers that capture more complex connections among predictor variables and the outcome of interest [4]. Natural language processing refers to the branch of computer science concerned with allowing computers to understand and generate text and spoken words like humans can [4]. The recent introduction of ChatGPT popularized a powerful modality that combines natural language processing and machine learning into large language models [5]. These are notable for their ability to achieve general-purpose language understanding and generation. Language models acquire these abilities by using massive amounts of data to learn billions of parameters during training and consuming ample computational resources during training and operation. First, we discuss ethical considerations in AI models to help pain physicians better critique such models. Second, we review some recent work related to using AI to predict postoperative opioid outcomes and opioid use disorder, as well as the use of large language models to track opioid-related outcomes from clinical text. Finally, we discuss future steps to improve AI in opioid management.

### Human and Animal Rights

All reported studies/experiments with human or animal subjects performed by the authors have been previously published and complied with all applicable ethical standards (including the Helsinki declaration and its amendments, institutional/national research committee standards, and international/national/institutional guidelines).

### Ethical Considerations for Predictive Models

In general, an important consideration when discussing predictive models in healthcare is related to their trustworthiness. For the purpose of this review paper, can the models predicting opioid-related outcomes provide fair, accurate, and generalizable predictions? Trustworthiness is vital to transitioning predictive models into real-time clinical workflow. Thus, this raises ethical concerns in AI that should be critiqued when determining overall trustworthiness. Notable domains in ethics as it relates to AI include, but are not limited to, (1) reproducibility and generalizability, (2) fairness and bias, and (3) transparency and explainability. (6, 7) Reproducibility and generalizability refer to whether predictive models will maintain their performance to a similar degree when tested on a separate patient population. Several opioid prediction models have been published that only report performance on a single dataset; thus, their generalizability is unclear. Fairness and bias are related to whether models have similar accuracy (or other model metrics) across various patient cohorts (e.g., racial, ethnic, sex/gender, etc.). Models with algorithmic bias may perpetuate disparities in care based on social groups and thus must be addressed to demonstrate trustworthiness. Finally, transparency refers to the authors’ clearness of model design, data processing, and model testing. Explainability may refer to providing explanations of why a model would make a prediction (e.g., the patient has the following risk factors that put them at higher risk of opioid addiction). (6, 7) The end-user for predictive models includes clinicians, and for these and other stakeholders to rely on AI for opioid outcomes, it is important that trustworthiness is established. In this review paper, not only do we discuss some of the current literature around predicting various opioid-related outcomes, but we also briefly point out the next steps to improve trustworthiness of these AI models prior to real-time use in clinical workflow.

### Predicting Persistent and Chronic Postoperative Opioid Use

Surgical patients may have persistent postoperative opioid use, especially in orthopedic surgery [8]. It is defined as the continued use of opioids for a certain period after surgery, which can be 2–3 months afterward [9]. Machine learning-based predictive models have been reported for spine surgery [10–12], knee arthroplasty [13–16], hip arthroplasty [17], arthroscopic joint surgery [18–21], outpatient surgery, (20, 21) and mixed surgical populations. (22, 23) The Stopping Opioids After Surgery score was described as a risk score for sustained opioid use after surgery (defined as sustained prescription opioid use for six months following surgery) [22]. This logistic regression-based model used claims data from TRICARE (the insurance program of the US Department of Defense) that included ~ 90,000 patients undergoing 1 of 10 common surgeries from 2005 to 2014. Prior opioid exposure was the strongest predictor for sustained opioid use. Hajouji et al. describe the development of machine learning-based models for predicting opioid-related adverse outcomes (e.g., opioid abuse, dependence, and overdose) 6 months after surgery using data from ~ 100,000 patients from Medicaid data [23]. A random forest approach achieved the best results, and analysis of feature importance demonstrated the most impactful predictors, such as cumulative daily opioid use, opioids at discharge, and depression.

Persistent opioid use after total knee arthroplasty has been reported to range from 7 to 24%.^13–14,16^ One study analyzed ~ 9,000 patients who underwent primary total knee arthroplasty, in which 7.2% experienced persistent opioid use beyond 90 days. Multiple machine learning models were tested, demonstrating high predictive accuracy, with areas under the receiver operating characteristics curve exceeding 0.80 [13]. Another study focused on predicting persistent opioid use post lower extremity joint arthroplasty, where rates were found to be around 23%. Key predictors included factors such as postoperative day one opioid consumption, body mass index, age, and preoperative opioid use. Using ensemble machine learning techniques and applying oversampling techniques for imbalanced data improved model performance [14]. In another study, about 10% of the study cohort turned into persistent opioid users, with baseline opioid use, demographics (age, gender, race), and medication use (benzodiazepines, anxiolytics, antidepressants) identified as significant predictors [16]. 

Collectively, these studies underscore the critical role of predictive modeling in identifying patients at risk for prolonged opioid use after knee arthroplasty. By leveraging such models, healthcare providers can implement individualized preoperative counseling and tailor perioperative pain management strategies, thereby reducing the risks associated with prolonged opioid use and enhancing patient recovery.

Persistent opioid use after spine surgery has been reported to range from 5% to higher than 50%, depending on the specific surgical population studied [10–12]. In a study with ~ 19,000 previously opioid-naïve patients who underwent surgery for lower back and lower extremity pain, about 5% met the criteria for long-term opioid use [10]. The models incorporated demographics, clinical comorbidities, preoperative opioid use, as well as 30-day postoperative opioid use. The machine learning models demonstrated area under the receiver operating characteristics curve (AUC) > 0.80, in which the most robust predictors were high preoperative opioid use and number of days and dosage increase with active opioid prescriptions postoperatively. Another study performed external validation on a Taiwanese population of a machine learning model predicting prolonged opioid prescriptions after lumbar disc herniation surgery that was developed on patient data from the United States [11]. The algorithm had good discriminative abilities despite differences in training and validation demographics. In another study with ~ 3,000 patients undergoing major spine surgery (not restricted to a single type of spine surgery), 46.3% were identified as having persistent opioid use [12]. Various machine learning models were developed, and the balanced random forest algorithm performed best based on the AUC and F1 scores. The features with the highest impact on model performance were age, preoperative opioid use, preoperative pain scores, and body mass index.

Identifying at-risk patients early on for persistent opioid use has several potential benefits, including providing a more concise allocation of resources that can help manage/monitor such patients. This includes services such as transitional pain clinics [24] or more specialized regional anesthesia techniques, such as cryo-neurolysis or peripheral neuromodulation. (25, 26) In addition, it can help model service allocations and guide Estates and the Federal government in drafting and addressing population and public health.

### Predicting Opioid Use Disorder and Overdose

The economic cost of the opioid epidemic is estimated to be tens of billions of dollars per year [27]. The US healthcare system is inextricably linked with the crisis, harnessing massive amounts of resources to treat patients with opioid use disorder [28] while also utilizing opioids as part of medically indicated pain reduction therapy. This interplay can result in the exposure of previously naive patients to opioids, which increases the risk of dependence. In one review, up to 75% of heroin users reported non-medical prescription opioid use as the primary impetus for their current dependence [29]. Another report found roughly 40% of opioid overdose fatalities involved prescription opioids [27]. 

Precision medicine is a disease treatment and prevention approach considering individual factors, including genetics, social environment, lifestyle, and clinical characteristics [30]. No one factor has been shown to account reliably for a large percentage of opioid use disorder risk – thus, risk stratification should be considered based on a constellation of factors that may play a role [31]. Opioid use disorder is a complex neurobiological disorder influenced by a myriad of genetic, environmental, and physiological characteristics. To effectively implement a precision medicine-based approach to preventing opioid use disorder, it is vital to develop tools that may identify patients early who are at high risk for dependence and to include multifaceted data in the analysis.

Several machine learning-based models have been described to predict an individual’s propensity for opioid use disorder [32–40] and opioid overdose [41–46]. Understanding the differences between how these models were designed is essential to highlight. For example, Dong et al. described using a long short-term memory model, a deep learning model incorporating data in a time series that incorporated electronic health record (EHR) data to predict opioid use disorder [32]. The features used in the model included the last five encounters (including diagnosis codes, medications, lab tests, clinical events, and demographics) before the target encounter (diagnosis of opioid use disorder). This approach outperformed other machine learning models used in the study. Kashyap et al. leveraged the MIMIC-III database (a single institution dataset with granular EHR data essentially consisting of critically ill patients) to train deep learning models to process structured (e.g., diagnosis codes, demographics, etc.) and unstructured data (e.g., clinical notes) to predict opioid prescription and opioid use disorder [33]. Model performance was appropriate. However, the disadvantage of this model was that it was predicted, based on a relatively short instance of time, if a patient would have a diagnosis of opioid use disorder during their intensive care unit stay. Therefore, it does not predict the risk of developing opioid use disorder longitudinally. Hastings et al. leveraged an administrative database based in Rhode Island to build machine-learning models to predict adverse opioid-related outcomes [45]. The model was trained on retrospective observations of Medicaid recipients who were prescribed an opioid. Features with the most vital importance included primary language, race/ethnicity, age, household size, and quarterly wages, to name a few.

Lo-Ciganic et al. described developing a machine-learning model to predict opioid overdose and external validation using a separate population [46]. Specifically, the models were developed to predict a 3-month risk of opioid overdose using training data from Pennsylvania Medicaid data and subsequently validated with later years of Pennsylvania Medicaid data as well as data from Arizona. Using data from Medicare beneficiaries and claims explicitly related to inpatient and emergency department opioid overdose episodes, machine learning algorithms were reported related to predicting overdose three months after initiation of prescription opioids [44]. The study comprised approximately 600,000 patients, each with about ~ 300 predictor candidates (patient, practitioner, and regional factors). Models demonstrated an excellent area under the receiver operating characteristics curve but a poor area under the precision-recall curve metrics. These studies demonstrate the promise AI has for early prediction of opioid use disorder and overdose, which may aid in precision medicine focused on the identification of high-risk patients to allocate resources more effectively.

### Tracking Opioid-Related Diagnoses from Clinical Text

Another application for AI models in the opioid realm includes surveilling medical records, potentially in real-time, to screen for opioid-related conditions, such as persistent postsurgical opioid use. Such approaches can rely on clinical notes rather than structured data (e.g., diagnosis codes), as the latter may not be up-to-date. AI-driven surveillance systems can potentially monitor opioid use patterns in real time and identify unusual prescribing behaviors and potential misuse. These systems can utilize electronic health records, prescription drug monitoring programs, and social media data to provide comprehensive oversight. Such surveillance helps in the early detection of abuse and intervention, thereby reducing the incidence of opioid use disorder [47]. 

Natural language processing and large language model approaches have been described to detect opioid use disorder [48–50] and persistent postsurgical opioid use [51–55]. Example use cases were reported related to identifying persistent opioid use after spine surgery [51] and total joint arthroplasty [52]. In these studies, persistent opioid use was defined as using opioids more than two months after surgery. The EHR may not routinely nor accurately record persistent opioid use in the form of structured data points, such as diagnosis codes or medication prescriptions (i.e., the presence of a prescription does not always mean the patient is actively consuming opioids). However, it may often be reported in outpatient surgical notes. These studies utilized large language models to process outpatient surgical follow-up notes to identify which patients were continuing to use opioids more than two months after surgery. Such AI-driven surveillance tools may aid clinicians in tracking persistent postoperative opioid use.

## Summary/Conclusion

What we are lacking in the literature for AI models and opioid outcomes is prospective validation studies to demonstrate both reproducibility and generalizability. Furthermore, more AI models should also report accurate/false favorable/negative rates among different social cohorts to indicate the presence of bias. Both are challenging feats but essential for gaining trustworthiness with AI and the end-user. Addressing these ethical issues is vital, mainly since conditions such as opioid use disorder may be represented in vulnerable populations. Furthermore, inaccurate predictions may lead to mistreating patients that are falsely identified as high or low risk.

Demonstrating generalizability is essential for enhancing the trustworthiness of AI models in practice. For example, a predictive model developed at DeepMind identified patients at high risk for acute kidney injury up to 48 h in advance [56]. The model was developed using training data from US Veterans, which comprised 94% male. When externally validated in a large academic hospital setting, poor discrimination of acute kidney injury was reported, especially among the female population [57]. Another example is the Epic Sepsis Model, a proprietary sepsis prediction model implemented at hundreds of US hospitals [58]. When externally validated at the University of Michigan, Ann Harbor (with nearly 40,000 patients), the Epic Sepsis Model demonstrated poor discrimination and calibration in predicting the onset of sepsis [59]. The key learning points here include the importance of proving generalizability, especially with opioid use outcomes. This can be done by validating reported models in the literature with clinicians’ institutional data and adjusting model features to improve generalizability with their population.

Addressing algorithmic bias is also critical for AI models. It is vital that models also report true and false favorable/negative rates for outcome predictions across various social groups, which could be based on race, ethnicity, or sex, for example. This has been a limited practice in AI models related to opioid use outcomes in the literature. There are several ways to improve fairness and bias. Ideally, AI models should be trained on diverse datasets to avoid biases that could lead to unequal treatment outcomes. They must also be continuously evaluated and updated to ensure they remain fair and effective across different patient populations [47]. Incorporation of social determinants of health is also essential to address algorithmic bias. AI can be leveraged to identify and address these determinants by analyzing data related to socioeconomic status, education, employment, social support, and neighborhood environments. Incorporating SDoH into AI models may enhance their predictive accuracy and ensure more comprehensive and equitable healthcare interventions. For example, individuals in economically disadvantaged areas with limited access to healthcare or social support could be more vulnerable to opioid misuse. By integrating these factors into predictive models, AI can help healthcare providers target interventions more effectively to those most in need [47]. 

Ethical considerations for AI models may lead to many generalizable uses for improving pain and opioid outcomes. AI can tailor personalized pain management plans to individual patients by analyzing various data points. For instance, reinforcement learning algorithms can personalize patient interactions, optimizing treatment strategies based on their responses and reported pain levels. This personalized approach may improve pain management outcomes and conserve healthcare resources, such as counselor time [47, 60]. AI models can identify patients at risk of developing opioid use disorder before they begin opioid therapy. By analyzing patient histories and patterns, these models can provide healthcare providers with critical insights, enabling them to take preventive measures, such as prescribing alternative therapies or implementing closer monitoring [61]. AI can help reduce inappropriate opioid prescriptions by providing decision support to clinicians. By evaluating patient data and clinical guidelines, AI systems may recommend the most appropriate pain management strategies, thus minimizing unnecessary opioid exposure. This approach aligns with the Center for Disease Control and Prevention’s recommendations for opioid prescribing, which emphasize the need for careful patient selection and dosing [61]. In acute pain management, AI can potentially predict the optimal opioid dosage needed for individual patients, reducing the risk of over-prescription. Studies have demonstrated that AI algorithms may estimate opioid requirements based on patient-specific factors such as age, weight, medical history, and type of surgery [62]. 

The successful integration of AI into pain practice requires training for healthcare providers and developing user-friendly interfaces. AI tools should complement, not replace, the clinical judgment of healthcare professionals, providing support and enhancing their ability to deliver personalized care. AI holds significant promise in enhancing the management of acute and chronic opioids, which may offer tools to help optimize dosing, predict addiction risks, and personalize pain management strategies. By harnessing the power of AI, healthcare providers can potentially improve patient outcomes, reduce the burden of opioid addiction, and contribute to solving the opioid crisis. Future research should focus on refining AI algorithms, addressing ethical concerns, and integrating these technologies into clinical practice to fully realize their potential.

## Key References

- Simpson S, Zhong W, Mehdipour S, et al. Classifying high risk patients for persistent opioid use after major spine surgery: A machine learning approach. *Anesth Analg.* 2024 Oct 1;139(4):690–699.
  - In this study, machine learning models using oversampling techniques were developed to predict persistent opioid use following major spine surgery. The most impactful predictors identified were age, preoperative opioid use, preoperative pain scores, and body mass index. The balanced random forest was found to be the most effective model for this data.
- Lo-Ciganic WH, Donohue JM, Yang Q, et al. Developing and validating a machine-learning algorithm to predict opioid overdose in Medicaid beneficiaries in two US states: a prognostic modelling study. Lancet Digit Health. 2022;4(6):e455-e465. 10.1016/S2589-7500(22)00062-0.
  - In this study, the authors developed a machine learning algorithm to predict 3-month risk of opioid overdose using Pennsylvania Medicaid data and externally validated it in two data sources (i.e., later years of Pennsylvania Medicaid data and data from a different state). The demonstrated that the models predicted opioid overdose performed well on external datasets and may be a valuable (and validated) model for opioid overdose risk prediction in Medicaid beneficiaries.

## Data Availability

No datasets were generated or analysed during the current study.
